# InCoB2010 - 9^th ^International Conference on Bioinformatics at Tokyo, Japan, September 26-28, 2010

**DOI:** 10.1186/1471-2105-11-S7-S1

**Published:** 2010-10-15

**Authors:** Christian Schönbach, Kenta Nakai, Tin Wee Tan, Shoba Ranganathan

**Affiliations:** 1 Department of Bioscience and Bioinformatics, Kyushu Institute of Technology, Fukuoka 820-8502, Japan; 2 Laboratory of Functional Analysis in silico, Human Genome Center, The Institute of Medical Science, The University of Tokyo, Tokyo 108-8639, Japan; 3 Department of Biochemistry, Yong Loo Lin School of Medicine, National University of Singapore, 8 Medical Drive, Singapore 117597; 4 Department of Chemistry and Biomolecular Sciences and ARC Centre of Excellence in Bioinformatics, Macquarie University, Sydney NSW 2109, Australia

## Abstract

The International Conference on Bioinformatics (InCoB), the annual conference of the  Asia-Pacific Bioinformatics Network (APBioNet), is hosted in one of countries of the Asia-Pacific region. The 2010 conference was awarded to Japan and has attracted more than one hundred high-quality research paper submissions. Thorough peer reviewing resulted in 47 (43.5%) accepted papers out of 108 submissions. Submissions from Japan, R.O. Korea, P.R. China, Australia, Singapore and U.S.A totaled 43.8% and contributed to 57.4% of accepted papers. Manuscripts originating from Taiwan and India added up to 42.8% of submissions and 28.3% of acceptances. The fifteen articles published in this *BMC Bioinformatics* supplement cover disease informatics, structural bioinformatics and drug design, biological databases and software tools, signaling pathways, gene regulatory and biochemical networks, evolution and sequence analysis.

## Introduction

InCoB (International Conference on Bioinformatics) is the official conference of the Asia-Pacific Bioinformatics Network (APBioNet) http://www.apbionet.org/. Since the inaugural conference in Bangkok in 2002, InCoB developed into one of the largest bioinformatics conferences in the Asia-Pacific region [1]. We attribute the growth in part to publishing submissions as research articles in the conference supplement of a PubMed-indexed open-access journal with a reasonable impact factor. Since 2006 InCoB has published 82 articles in *BMC Bioinformatics* that were cited 436 times (as of May 2010). Three years later (InCoB2009 Singapore), the increasing number of submissions necessitated the addition of a second InCoB supplement in *BMC Genomics*[1].

InCoB annual conferences showcase the latest research and technologies in all areas of bioinformatics. This year's collaboration with Chem-Bio Informatics Society (CBI) http://www.cbi.or.jp of Japan and International Immunomics Society (IIMMS) http://www.iimms.org/ and support of the Japanese Society for Bioinformatics http://www.jsbi.org attracted a diverse spectrum of submission including papers on synthetic biology, biocomputing, systems biology, computational immunology/vaccinology and disease informatics. Some of the topics will be covered in depth on the third conference day when IIMMS and CBI hold their 3^rd ^Conference of Basic and Clinical Immunogenomics and Immunomics and CBI Workshop on Synthetic Biology, Molecular Robotics and Translational Bioinformatics.

## Submissions and review

**Figure 1:**
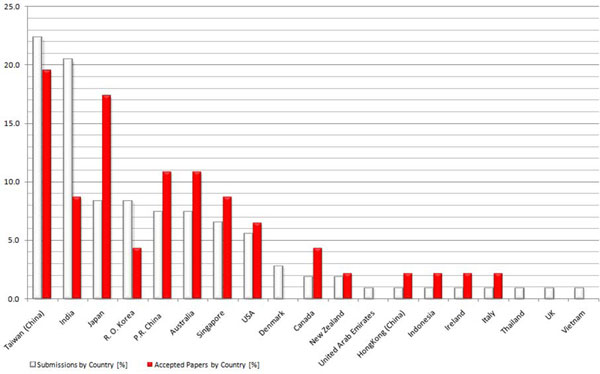
**Submitted *vs. *accepted articles by country**.

Of the 108 submissions received, we accepted 15 articles for *BMC Bioinformatics*, 25 for *BMC Genomics*[2] and six for *Immunome Research*[3], an independent BMC journal that publishes bioinformatics-driven immunology research, and one for *IPSJ Transactions on Bioinformatics*. The submitted articles originated from 19 countries with Taiwan and India contributing almost 43% of submissions and 28% of accepted articles (Fig. 1). Taiwan's proximity to Japan and the success of IncoB2008 [4] in Taipei may explain this very high submission rate. The lower acceptance rate of manuscripts from India compared to that of Taiwan is the result of several submissions that were outside the scope of the three concerned journals. Each submission was peer-reviewed by three PC members and/or sub-reviewers, excepting two, both of which had excellent scores from the reviewers, verified by the editors. In an attempt to raise the quality and impact of manuscripts, we introduced a second round of reviews for revised submissions that were originally judged as borderline papers. Two submissions underwent three rounds of revisions and reviewing before being accepted. We wish to thank the 83 Program Committee members and 56 sub-reviewers (Additional File 1) for lending their precious time, providing objective comments and responding promptly to our multiple review requests.

The challenges and returns of applying bioinformatics research to the areas of disease informatics, structural bioinformatics and drug design, biological databases and tools, systems biology focussing on biological networks and signaling pathways, and sequence analysis are highlighted in this issue.

## Disease informatics

Homozygosity mapping is a standard method to identify recessive disease-causing genes if the data size is sufficiently large. Huqun *et al. *[5] developed a homozygosity mapping tool (qHomozygosityMapping) that can identify recessive disease-causing genes using high-density single nucleotide polymorphism arrays from very small sample sizes. The success of this new approach was confirmed the power of this algorithm using data from six patients with a rare autosomal recessive disease in Japan, the Siiyama-type α1-antitrypsin deficiency. Pennisi and co-workers [6] introduce hybrid agent-based models that simulate the vaccine-elicited immune system response against murine lung metastases. The models aim to maximize the reduction of metastases while minimizing the number of vaccinations.

## Structural bioinformatics and inhibitory drug design

In an analysis of intrinsically disordered domains (IDDs) in innate-immune-related proteins in mouse and generic human proteins Teraguchi and co-workers [7] clustered IDDs into sub-groups and associated the clusters with various ordered domains. Ligand- and structure-based virtual screening approaches are typical methods in identifying inhibitor drug candidates. Nagarajan *et al. *[8] applied 3D-QSAR, recursive partitioning, and docking filters to identify hit compounds that inhibit NIKKβ, an important target in anti-inflammatory drug research. Usman and Wulandari [9] report the docking results of two histone deacetylase family inhibitors. Pharmacological properties gleaned from the result might be useful for designing new inhibitors targeting cervical cancer.

## Biological databases and tools

Chen *et al. *[11] have developed DoDo and efficient method for detecting orthologous genes by using domain information. DoDo outperforms InParanoid [11]. Effectors of Type III Secretion System database (T3SSdb) contains annotations that permit the effective design of prediction models to discover new effector candidates [12].

## Systems biology: biological networks and signaling pathways

Protein-protein interactions (PPI) are essential features of many cellular processes. In a large-scale topological analysis of *Escherichia coli *non-coding small RNA targets in transcription regulatory and PPI networks, Wu *et al. *[13] identified general modular relationships among the regulatory networks. The findings of experimental and predicted OxyS targets in response to oxidative stress are discussed as proof of concept. Wu and colleagues [14] present a new two-step method that integrates various biological and computational sources using Bayesian statistics or support vector machine methods to construct reliable yeast PPI networks. Some interesting differences in the subcellular locations of proteins between human PPI and metabolic networks were reported by Kumar and Ranganathan [15]. The authors analyzed metabolic pathways, from the viewpoint of subcellular localization of proteins in the networks. In a detailed study of high-mobility group box-1 protein (HMGB1) signaling pathway Gong *et al. *[16] introduced a computational model that simulates the cross-talk between HMGB1 and p53, NFκB, TNF and other signaling pathways that are implicated in cancer. The last paper in the network category takes us through the sensitivity analysis of large biological reaction using time-delayed differential equations (DDEs) [17]. The authors demonstrated the efficacy of their DDEs in numerical interacellular kinetic simulations of the cardiovascular control system and the TNFα signal transduction network.

## Sequence analysis

Three papers show the power of computational biology in addressing questions of general biological interest. Okamura et al. [18] present a new method of analyzing methylation patterns. Their analysis of 26 deuterosome genomes revealed a directional transition of methylation patterns from cephalochordates to the urochordate-vertebrate split. The results imply that the mammalian global DNA methylation pattern arose gradually from fractional patterns. Saito *et al. *[19] extended a previously published base-pairing profile local alignment (BPLA) kernel method for single sequences to predict non-coding RNAs from structure-based aligned sequences of large datasets. BPLA method showed superior prediction accuracy and robustness against errors. Metagenomic sequence analysis requires the taxonomic binning of sequenced reads. Ghosh et al. [20] have improved their earlier algorithm by addressing the binning quality and speed associated.

## Conclusion

Conference attendance and networking can provide a rich source of inspirations and excellent opportunities to exchange information on a broad spectrum of cutting-edge biological and computational topics. To advance bioinformatics research, it is important to continuously engage in information exchange. Towards this end, we provided to one author of each article a complimentary one-year membership in the International Society of Computational Biology (ISCB), and hope to see them again at 10^th ^InCoB in Kuala Lumpur in 2011 or at one of the ISCB conferences.

## Competing interests

The authors declare that they have no competing interests.

## Authors' contributions

CS and KN are the Conference Co-chairs and contributed equally the overall organization of InCoB 2010. TWT (APBioNet Secretariat) and SR (President, APBioNet and Publication Committee Chair) provided advice in organizing InCoB2010 and supported post-acceptance manuscript processing.

## Supplementary Material

Additional file 1 **List of Program Committee members and subreviewers**. Click here for file
