# Supplementary material for: InCoB2010 - 9th International Conference on Bioinformatics at Tokyo, Japan, September 26-28, 2010
Source: BMC Bioinformatics. 2010 Oct 15;11(Suppl 7):S1. doi: 10.1186/1471-2105-11-S7-S1 (PMC2957677; doi:10.1186/1471-2105-11-S7-S1)
Supplement: Additional file 1 — List of Program Committee members and subreviewers. [file 1471-2105-11-S7-S1-S1.pdf]

Additional File 1: List of Program Committee members and subreviewers

InCoB2010 – 9th International Conference on Bioinformatics at Tokyo, Japan, September 26-28, 2010

by

Christian Schönbach, Kenta Nakai, Tin Wee Tan and Shoba Ranganathan

InCoB2010 - 9th International Conference on Bioinformatics
Tokyo, Japan, September 26-28, 2010

in conjunction with

IIMMS 3rd Conference of Basic and Clinical Immunogenomics and Immunomics
and
CBI Workshop on Synthetic Biology, Molecular Robotics and Translational Bioinformatics
Tokyo, September 28, 2010

InCoB Program Committee (in alphabetical order)

Tatsuya Akutsu, Kyoto University, Japan

Peter Antal, Budapest University of Technology and Economics, Hungary

Vladimir Bajic, King Abdullah University of Science and Technology, Saudi Arabia

Christopher J. O. Baker, University of New Brunswick, Canada

Sergio Baranzini, University of California, San Francisco, USA

Alex Bateman, The Wellcome Trust Sanger Institute, UK

Jonas Bergman Laurila, University of New Brunswick, Canada

Zhiwei Cao, Tongji University, P.R. China

Filippo Castiglione, IAC "M. Picone", Italy

Alan Christoffels, South African National Bioinformatics Institute, South Africa

Adrian Cootes, Macquarie University, Australia

Anne D Groot, EpiVax Inc., USA

David S. DeLuca, Dana-Farber Cancer Institute, Harvard University, USA

Frank Eisenhaber, Bioinformatics Institute, A*STAR, Singapore

Darren Flower, Aston University, UK

Ge Gao, Center for Bioinformatics, Peking University, P.R. China

Takashi Gojobori, National Institute of Genetics, Japan

Susumu Goto, Kyoto University, Japan

M. Michael Gromiha, Computational Biology Research Center, AIST, Japan

Taizo Hanai, Kyushu University, Japan

Chia-Lang Hsu, National Yang-Ming University, Taiwan

Wen-Lian Hsu, Academia Sinica, Taiwan

Chun-Hsi Huang, University of Connecticut, USA

Mitsuhiro Itaya, Keio University, Japan

Hideo Iwasaki, Waseda University, Japan

Javed Mohammed Khan, Macquarie University, Australia

Daisuke Kiga, Tokyo Institute of Technology, Japan

Akira Kinjyo, Osaka University, Japan

Kengo Kinoshita, Tohoku University, Japan

Tetsuya Kobayashi, University of Tokyo, Japan

Akihiko Konagaya, Tokyo Institute of Technology, Japan

Hiroyuki Kurata, Kyushu Institute of Technology, Japan

Igor V. Kurochkin, Bioinformatics Institute, A*STAR, Singapore

Chih Lee, University of Connecticut, USA

Marie-Paule Lefranc, Université Montpellier 2, CNRS, IFR3, France

Ole Lund, Technical University of Denmark, Denmark

Hiroshi Mamitsuka, Kyoto University, Japan

Satoshi Murata, Tohoku University, Japan

Hideo Matsuda, Osaka University, Japan

Santo Motta, University of Catania, Italy

H.A. Nagarajaram, Centre for DNA Fingerprinting and Diagnostics, India

Kenta Nakai (PC Co-Chair), University of Tokyo, Japan

Haruki Nakamura, Osaka University, Japan

See-Kiong Ng, Institute for Infocomm Research, Singapore

Jun Ni, The University of Iowa, USA

Takenao Ohkawa, Kobe University, Japan

Masahiro Okamoto, Kyushu University, Japan

Yasushi Okazaki, Saitama Medical University, Japan

Ashwini Patil, University of Tokyo, Japan

Nikolai Petrovsky, Flinders Medical Centre, Australia

Jagath C. Rajapakse, Nanyang Technological University, Singapore
Shoba Ranganathan, Macquarie University, Australia
Timothy Ravasi, King Abdullah University of Science and Technology, Saudi Arabia
Rintaro Saito, Keio University, Japan
Yasubumi Sakakibara, Keio University, Japan
Christian Schönbach (PC Co-Chair), Kyushu Institute of Technology, Japan
Masakaso Sekijima, Tokyo Institute of Technology, Japan
Mohammad Tabrez Anwar Shamim, Centre for DNA Fingerprinting and Diagnostics, India
Kiyotaka Shiba, Japanese Foundation for Cancer Research, Japan
Tetsuo Shibuya, University of Tokyo, Japan
Kazuyuki Shimizu, Kyushu Institute of Technology, Japan
Worachart Sirawaraporn, Mahidol University, Thailand
Daron M Standley Osaka University, Japan
Durai Sundar, Indian Institute of Technology Delhi, India
Toyotaro Suzumura, Tokyo Institute of Technology, Japan
Yoshihiro Taguchi, Chuo University, Japan
Takao Takai-Igarashi, Tokyo Medical and Dental University, Japan
Yoichi Takenaka, Osaka University, Japan
Tin Wee Tan, National University of Singapore, Singapore
Hiroshi Tanaka, Tokyo Medical and Dental University, Japan
Joo Chuan Tong, Institute for Infocomm Research, A*STAR, Singapore
Tetsuro Toyoda, RIKEN BASE (Bioinformatics And Systems Engineering), Japan
Tatsuhiko Tsunoda, RIKEN Center for Genomic Medicine, Japan
Hiroki R Ueda, RIKEN Center for Developmental Biology, Japan
Chandra Verma, Bioinformatics Institute, A*STAR, Singapore
Hiroshi Wako, Waseda University, Japan
Dennis Paul Wall, CBMI, Harvard University, USA
Limsoon Wong, National University of Singapore, Singapore
Ueng-Chang Yang, National Yang-Ming University, Taiwan
Masayuki Yamamura, Tokyo Institute of Technology, Japan
Tetsuya Yomo, Osaka University, Japan
Kei Yura, Ochanomizu University, Japan
Guanglan Zhang, Dana-Farber Cancer Institute, Harvard University, USA

InCoB2010 Sub-reviewers

We wish thank the following sub-reviewers for contributing with their expertise to the review process:
Shandar Ahmad, Matthew Ardito, Masanori Arita, Daniel Berrar, David Blair, Raffaele Calogero, Qiang Chen, Jose C. Clemente, Adrian Cootes, Jike Cui, Todd DeLuca, Irini Doytchinova, Takaho Endo, Martin Frith, Andre Fujita, Seiya Imoto, Takashi Ishida, Todd Johnson, Jae-Yoon Jung, Takeya Kasukawa, Shuichi Kawano, Asif M. Khan, Pouya Khankhanian, Ryotaro Koike, Tomokazu Konishi, Hidetoshi Kono, William Martin, Hideaki Mizuno, Somay Y. Murayama, Shivashankar Nagaraj, So Nakagawa, Hiroshi Nakashima, Morten Nielsen, Itoshi Nikaido, Hideki Noguchi, Thomas Nordahl Peterson, Takeshi Obayashi, Kohji Okamura, Noriaki Okimoto, Andrew Orr, Sung-Joon Park, Bent Petersen, Jaume Perez-Sanchez, Mark A. Ragan, Kengo Sato, Kenji Satou, Jun Sese, Masafumi Shionyu, Matsuyuki Shirota, Mikita Suyama, Yoshio Tateno, Hiroyuki Toh, Hachiya Tsuyoshi, Lawrence Wee, Chao Xie, and Tetsushi Yada.
